# Multimodal on-chip nanoscopy and quantitative phase imaging reveals the nanoscale morphology of liver sinusoidal endothelial cells

**DOI:** 10.1073/pnas.2115323118

**Published:** 2021-11-15

**Authors:** Ankit Butola, David A. Coucheron, Karolina Szafranska, Azeem Ahmad, Hong Mao, Jean-Claude Tinguely, Peter McCourt, Paramasivam Senthilkumaran, Dalip Singh Mehta, Krishna Agarwal, Balpreet Singh Ahluwalia

**Affiliations:** ^a^Department of Physics and Technology, Universitetet i Tromsø (UiT) The Arctic University of Norway, 9037 Tromsø, Norway;; ^b^Bio-photonics and Green Photonics Laboratory, Department of Physics, Indian Institute of Technology Delhi, New Delhi 110016, India;; ^c^Faculty of Health Sciences, Department of Medical Biology, Vascular Biology Research Group, UiT The Arctic University of Norway, Tromsø 9037, Norway;; ^d^Department of Clinical Science, Intervention and Technology, Karolinska Institutet, 17177 Stockholm, Sweden

**Keywords:** superresolution microscopy, bioimaging, on-chip nanoscopy, liver sinusoidal endothelial cells

## Abstract

Visualization of three-dimensional (3D) morphological changes in the subcellular structures of a biological specimen is a major challenge in life science. Here, we present an integrated chip-based optical nanoscopy combined with quantitative phase microscopy (QPM) to obtain 3D morphology of liver sinusoidal endothelial cells (LSEC). LSEC have unique morphology with small nanopores (50-300 nm in diameter) in the plasma membrane, called fenestrations. The fenestrations are grouped in discrete clusters, which are around 100 to 200 nm thick. Thus, imaging and quantification of fenestrations and sieve plate thickness require resolution and sensitivity of sub-100 nm along both the lateral and the axial directions, respectively. In chip-based nanoscopy, the optical waveguides are used both for hosting and illuminating the sample. The fluorescence signal is captured by an upright microscope, which is converted into a Linnik-type interferometer to sequentially acquire both superresolved images and phase information of the sample. The multimodal microscope provided an estimate of the fenestration diameter of 119 ± 53 nm and average thickness of the sieve plates of 136.6 ± 42.4 nm, assuming the constant refractive index of cell membrane to be 1.38. Further, LSEC were treated with cytochalasin B to demonstrate the possibility of precise detection in the cell height. The mean phase value of the fenestrated area in normal and treated cells was found to be 161 ± 50 mrad and 109 ± 49 mrad, respectively. The proposed multimodal technique offers nanoscale visualization of both the lateral size and the thickness map, which would be of broader interest in the fields of cell biology and bioimaging.

## Significance

We propose the integration of chip-based optical nanoscopy with high spatially sensitive quantitative phase microscopy to obtain three-dimensional (3D) morphology of liver sinusoidal endothelial cells (LSEC). LSEC contain large numbers of transcellular nanopores —“fenestrations”—in the plasma membrane, typically clustered in groups of 10 to 50 within areas called sieve plates. Determining the diameter and the height of fenestrated regions is an important indicator of a cell’s functionality, and these dimensions can be influenced by agents such as drugs. Our proposed multimodal microscope offers a solution for 3D nanoscale characterization of fenestration diameter and measurement of the optical thickness of the sieve plates.

Far-field optical nanoscopy techniques are frequently used to visualize subcellular structures in biological specimens by surpassing the diffraction limit. Optical nanoscopy encompasses a plethora of techniques, including stimulated emission depletion microscopy (1), structured illumination microscopy (SIM) (2), different variants of single-molecule localization microscopy (SMLM), such as photo-activated localization microscopy (3) and direct stochastic optical reconstruction microscopy (*d*STORM) (4), and intensity fluctuation–based techniques such as superresolution optical fluctuation imaging (5). These techniques can help detect subcellular structures (<200 nm) of biological specimens such as lipids, proteins, membrane structures, microtubules, and nucleic acids by specific fluorescence tagging (6). Each technique has respective advantages and disadvantages; for example, SIM has gained popularity for live-cell imaging due to its fast image acquisition time but at limited spatial resolution (7). *d*STORM, on the other hand, is slower but offers high resolution for characterization of viral proteins (8) and imaging actin filaments in mammalian cells (9, 10), for example. To reduce the complexity of the typical SMLM setup using a total internal reflection fluorescence (TIRF) configuration, a photonic chip-based optical nanoscopy system was recently proposed (11–13). In the chip-based system, a photonic integrated circuit is used to replace the usual free space optics for excitation. The collection, however, is done through free space optics. The main advantage of this configuration is the decoupling of excitation and collection pathways as well as miniaturization of the excitation light path of the system. In chip-based nanoscopy, the TIRF illumination is generated through the evanescent field of waveguides rather than using conventional high magnification and high numerical aperture (N.A.) TIRF lens. The evanescent field in waveguides can be generated over extraordinarily large areas, as it is only defined by the waveguide geometry. The waveguide geometry makes it possible to use any imaging objective lens to image arbitrarily large areas as compared to the traditional TIRF-based *d*STORM (12), which is limited by the field of view (FOV) of the TIRF lens.

Quantitative phase microscopy (QPM) is a label-free optical microscopy technique, which facilitates sensitive measurements of the refractive index and thickness of both biological specimens (14). Various QPM methods have been proposed so far for extracting optical phase and dynamics of biological cells (15–17). These techniques offer high phase sensitivity (spatial and temporal), transverse resolution, and high imaging speed (15). The spatial and temporal phase sensitivity of the QPM system is highly dependent on the illumination source and the type of interferometric geometry, respectively (17–19). For example, common path QPM techniques offer better temporal phase sensitivity, which can be used to measure membrane fluctuation of the cells (20). In addition, spatial phase sensitivity of the system can be improved by using low-coherence light sources (halogen lamps and light-emitting diodes [LED]) but requires phase-shifting techniques to utilize the whole FOV of the camera (21). A recent advancement in the QPM technique with superior resolution using structured illumination (22, 23) and three-dimensional (3D) information of the samples has been shown by measuring the phase across multiple angles of illumination. This technique facilitates tomography of various biological specimens such as red blood cells, HT29 cells, and bovine embryos (17, 24). Since the lateral resolution of the QPM technique depends on the N.A. of the objective lens, imaging beyond the diffraction limit (<200 nm) is still challenging and limits the study of subcellular structures. Therefore, it is useful to develop multimodality routes in which different microscopy methods can be utilized to provide complementary information about biological specimens such as liver sinusoidal endothelial cells (LSEC).

**Fig. 1.**
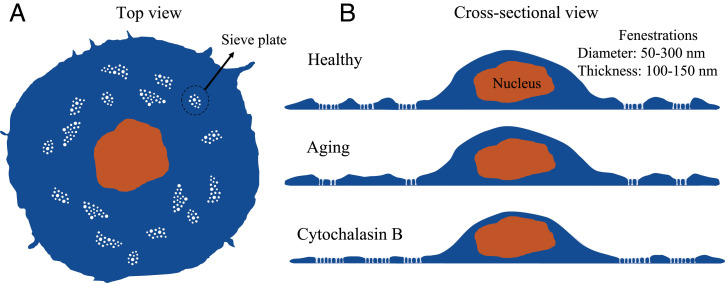
Top view (*A*) and cross-sectional view (*B*) of LSEC. LSEC have unique morphology, in which nanoscopic fenestrations are grouped in thin sieve plates. The diameter of fenestrations and thickness of sieve plates are below the diffraction limit of conventional optical microscopes. The number and size of fenestrations, but also LSEC thickness, can be affected by aging and in liver diseases. In vitro, the number of fenestrations can be increased using actin disrupting agents, such as cytochalasin B (27).

Fig. 1 depicts LSEC that contain large numbers of fenestrations. These transcellular nanopores vary in diameter from 50 to 300 nm, which is just below the diffraction limit of optical microscopy (25–27). Fenestrations are typically clustered in groups of 5 to 100 within areas called sieve plates (28). The porous morphology of LSEC acts as an ultrafilter between blood and the underlying hepatocytes, facilitating the bidirectional exchange of substrates between the interior of the liver and blood. For example, smaller viruses and drugs can pass this barrier, while blood cells are retained within the sinusoidal vessel lumen (25, 29). The typical thickness of sieve plates is around 100 to 150 nm (30), so fenestrations are consequently nanoscale sized in all three dimensions. As shown in Fig. 1, the fenestrations in sieve plates form openings through the entire LSEC cell body, and therefore TIRF illumination is ideally suited for imaging these structures. Determining the diameter and number of fenestrations, as well as the height of sieve plate regions, is important, as it can be affected by several drugs and conditions (31, 32). The loss of LSEC porous morphology, a process called defenestration, compromises the filtration properties of the liver, which may lead to atherosclerosis (33). Moreover, aging results in “pseudocapillarization,” whereby LSEC simultaneously lose fenestrations and become thicker (34) (Fig. 1). This is believed to be a main factor contributing to the age-related need to increase doses of drugs targeting hepatocytes (e.g., statins) that have to pass through the fenestrations (35). The number of fenestrations in vitro can be increased using actin disrupting agents such as cytochalasin B (27). This treatment decreases the height of LSEC outside of the nuclear area, which contributes to the formation of new fenestrations (36).

Here, we have developed a multimodal chip-based optical nanoscopy and highly sensitive QPM system to visualize the 3D morphological changes in LSEC. The proposed system decouples the light illumination path from the collection path and thus enables a straightforward integration of *d*STORM and QPM. The nanoscale phase sensitivity of the QPM technique is utilized to extract the optical thickness of sieve plates. Moreover, chip-based *d*STORM supports superresolution imaging down to 50 nm over an extraordinarily large FOV up to millimeter scale (12). Therefore, integration of *d*STORM and QPM allows superresolution imaging in the lateral dimension (with *d*STORM) and nanometric sensitivity in the axial direction (with QPM). In this work, we demonstrate the capabilities of the system by imaging LSEC with both diffraction-limited TIRF microscopy and *d*STORM. The fenestrations and sieve plates are observable with *d*STORM, and the average optical thickness of the sieve plate region is obtained using diffraction-limited QPM. Furthermore, we investigated the change in the interior morphology of sieve plates by treating the cell with cytochalasin B (10 μg/mL). The deficiency of lateral resolution of QPM was compensated by *d*STORM, which enabled us to localize the sieve plate regions containing subdiffraction-sized fenestration. Therefore, in the cell membrane regions distal from the nucleus, the 3D morphology of LSEC can be reconstructed reliably using our multimodal approach. The integrated system offers a combination of simultaneous functional and quantitative imaging of the cells with large FOV, providing a compact imaging platform with a potential for high-throughput morphological and nanometric imaging for specific biological applications.

## Experimental Details

### Working Principle of a Partially Spatially Incoherent Quantitative Phase Microscope.

**Fig. 2.**
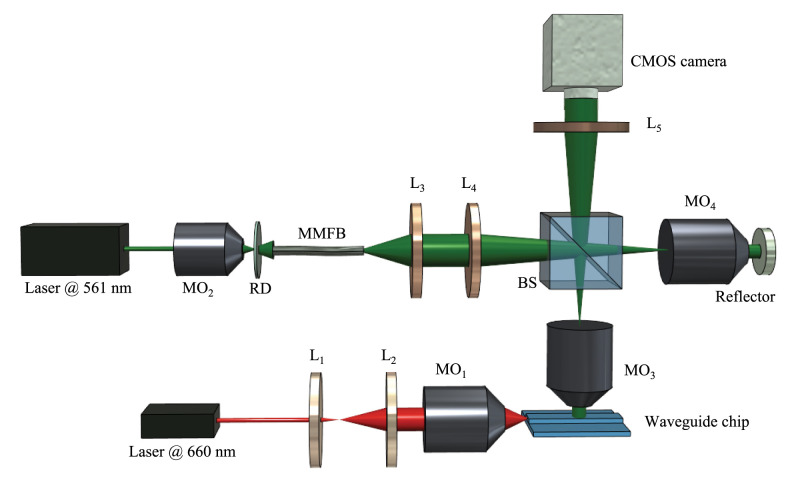
Diagram of integrated partially spatially incoherent QPM and chip-based nanoscopy system for the morphological imaging of LSEC. MO_1–4_: microscopic objective lens; RD: rotating diffuser; L_1–5_: lens; BS: beam splitter. The high intensity evanescent field is generated on top of the waveguide chip using a 660-nm Cobolt laser for single-molecule fluorescence excitation. The fluorescence signal is captured by an upright microscope, which is converted into a Linnik-type interferometer to perform QPM.

A diagram of the system in QPM and *d*STORM mode is shown in Fig. 2. A 660-nm laser (Cobolt Flamenco, λ = 660 nm) is coupled into the waveguide to generate the evanescent field on the waveguide surface for the *d*STORM experiment. A highly coherent 561-nm laser (Cobolt Jive, λ = 561 nm) is expanded by a microscopic objective and passes through the rotating diffuser followed by a multimode fiber (MMFB, M35L02 - Ø1000 µm; Thorlabs) for the phase imaging. The rotating diffuser and MMFB are used to generate spatial and temporal diversity to convert a highly coherent laser into a partially spatially coherent light source. It has been shown previously that the reduction of spatial coherence results in speckle-free images and improves the spatial phase sensitivity of the interferometry system (37–39). Therefore, partially spatially coherent sources (PTLS) can be utilized to extract the morphological changes of the thinnest biological specimens such as LSEC. The partially spatially coherent beam with ∼10 mW power is coupled into the Linnik-type QPM system. In the QPM system, light beams reflected from the sample and reference mirror interfere at the beam splitter plane. We used a 60×, 1.2 N.A. water immersion objective lens (Olympus) for all QPM measurements, meaning the best achievable lateral resolution is 270 nm. The two-dimensional (2D) interference pattern coded the information of the sample, which is further captured by the complementary metal oxide semiconductor (CMOS) image sensor (Hamamatsu ORCA-Flash4.0 LT, C11440-42U).

The 2D intensity distribution of the interferogram can be expressed as

[1] $$I\;\left(x,y\right)=a\left(x,y\right)+b\left(x,y\right)\text{cos}[2i\left(f_{x}x+f_{y}y+\phi \left(x,y\right)\right],$$

where *a*(*x,y*) and *b*(*x,y*) represent the background and the modulation terms, respectively. ${\text{f}}_{\text{x}}\text{x}$ and ${\text{f}}_{\text{y}}\text{y}$ are the spatial frequencies of the interference pattern along *x* and *y* directions, and ϕ (x,y) is the phase difference between the object and reference beam.

Standard Fourier transform analysis (40) and the Goldstein phase unwrapping algorithm (41) are used to extract the phase information of the specimens. The phase information is a combination of refractive index and thickness of the specimens and can be written as

[2] $$\phi \left(x,y\right)=\frac{2\pi }{\lambda }\times 2h\left(x,y\right)*\left\{n_{s}\left(x,y\right)-n_{0}\left(x,y\right)\right\},$$

where *λ* is the wavelength of incident light, *h* is the geometrical thickness of the specimen, *n_s_* and *n_0_* are the refractive indices of the specimen and surrounding medium, respectively, and an extra factor of 2 appears because the imaging is performed in the reflection mode. By reformatting the equation, an expression for the thickness of the sample can be derived:

[3] $$h\left(x,y\right)=\;\frac{\lambda *\phi \left(x,y\right)}{4\pi *\left\{n_{s}\left(x,y\right)-n_{0}\left(x,y\right)\right\}}.$$

### *d*STORM Imaging and Data Analysis.

*d*STORM imaging was performed through waveguide chip-based TIRF excitation. Once the sample was stained and the blinking buffer (9, 10) added, the chip was placed on the sample stage and held in place with a vacuum chuck. The excitation light was coupled from free space by end-fire coupling using a 50×, 0.5-N.A. objective lens (Olympus). The waveguides are multimoded, giving rise to an inhomogeneous excitation pattern. In order to achieve homogeneous illumination, the coupling objective was scanned along the input facet to average out the modes. Imaging was done with a Hamamatsu Orca scientific complementary metal oxide semiconductor camera with 30-ms exposure time. For TIRF images, the exposure time was increased to 100 ms and an average of ∼1,000 frames used. Approximately 20 mW input power was used for all images; however, the power was incrementally increased up to ∼60 mW toward the end of each imaging procedure to obtain additional localizations. The data were reconstructed using ThunderSTORM (42), a FIJI plugin. More details of this type of setup can be found in literature (11–13).

## Materials and Methods

### Chip Preparation.

**Fig. 3.**
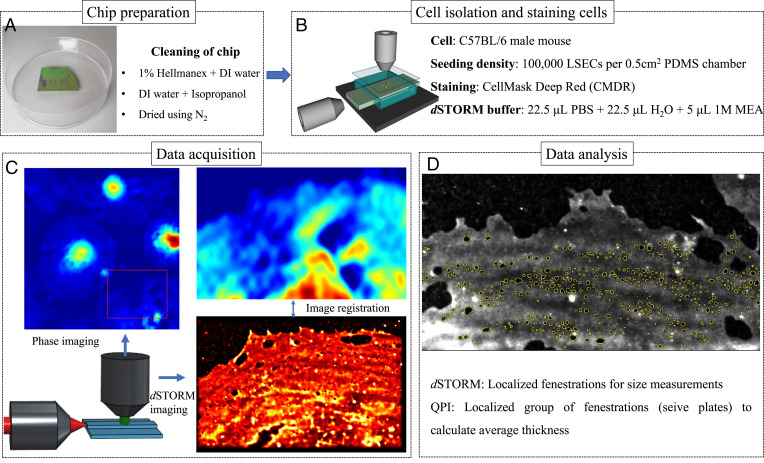
Workflow of the integrated QPM and on-chip nanoscopy system. (*A*) Si_3_N_4_ strip waveguides are cleaned thoroughly to perform all imaging experiments. (*B*) Cells are isolated on top of the chip within a restricted rectangular area created with PDMS. *C* and *D* show the data acquisitions and registration between quantitative phase imaging (QPI) and superresolution imaging to calculate the size of the fenestrations and the average thickness of the group of fenestrated areas in LSEC.

The workflow of the system to extract the optical thickness size of fenestrated areas in LSEC is shown in Fig. 3. All imaging in the present work was done using Si_3_N_4_ strip waveguides with varying widths of between 200 and 500 μm. The chips were fabricated using a previously described procedure (43). Before any sample preparation, the chips were thoroughly cleaned using a two-step process. First, the chips were submersed in a 1% Hellmanex in deionized (DI) water solution at 70 °C for 10 min and then rinsed with DI water, followed by submersion in isopropanol and rinsing in DI water again. Finally, the chips were dried using N_2_. A hollow rectangular chamber was created with polydimethylsiloxane (PDMS) and placed on the chip to restrict the cell attachment area.

### Cell Isolation and Seeding.

Cells were isolated from C57BL/6 male mice and cryopreserved Sprague Dawley male rats using a modified standard protocol (44). Briefly, perfusion of the liver with Liberase (Roche) was followed by low-speed differential centrifugation and then cell separation using superferromagnetic beads conjugated with the LSEC-specific antibody CD146 (MACS, Miltenyi Biotec). After isolation, the cells were seeded on chips precoated with human fibronectin and incubated in 5% CO_2_ at 37 °C in RPMI-1640 culture medium for 2 h. Seeding density was about 100,000 LSEC per 0.5 cm^2^ in a PDMS chamber. Selected samples were treated for 30 min with 10 μg/mL cytochalasin B (Sigma-Aldrich). Cells were fixed by 10 min incubation in 4% paraformaldehyde (PFA) in phosphate-buffered saline (PBS) and left in 1% PFA at 4 °C until imaging.

### Staining Protocol and Data Acquisitions.

The cells were stained with CellMask Deep Red (CMDR) and Vybrant DiD with the chips being rinsed thoroughly with PBS before staining. A 1:1,000 dilution of CMDR in PBS was added to the inside of the PDMS chamber and left to incubate for 10 min. For Vybrant DiD, a 1:200 dilution in PBS was added to the inside of the PDMS chamber for 20 min. The sample was then thoroughly rinsed with PBS again. Prior to imaging, a dSTORM buffer was prepared using 22.5 μL PBS, 22.5 μL H_2_O-based oxygen scavenger system solution (45), and 5 μL 1 M β-mercaptoethylamine (MEA). The sample was then rinsed thoroughly with PBS before the blinking buffer was applied and the sample area sealed off with a coverslip. Finally, the chip was placed under the microscope to acquire both interferometric and *d*STORM imaging. The extracted phase map and superresolution images are further registered to localize the fenestrated area and to calculate size and optical thickness of the sieve plates. Fenestrations were quantified using an intensity-based threshold method similar to the semiautomatic method described in ref. 46.

## Results and Discussion

**Fig. 4.**
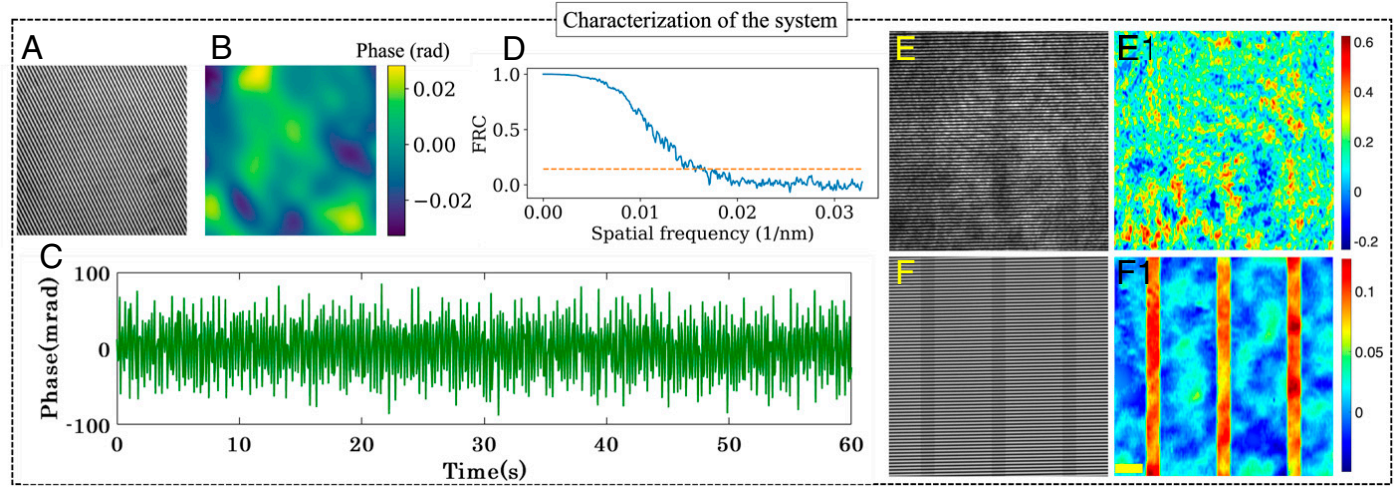
Noise and resolution characterization of the integrated QPM and *d*STORM system. (*A*) Interferogram captured by the QPM mode of the proposed setup on a standard mirror of λ/10 surface flatness. (*B*) SD of spatial phase in *A* demonstrating the spatial phase noise (±20 mrad) of the system. The color bar represents the phase map in radians. (*C*) Temporal noise (±38 mrad) of the phase microscopy system. (*D*) The 61-nm lateral optical resolution of the chip-based system was obtained on the sample using FRC. (*E* and *F*) Interferometric images of Si_3_N_4_ optical waveguides while using direct laser and PTLS, respectively. (*E1* and *F1*) Reconstructed full FOV phase maps in radians of an optical waveguide (H ∼8 nm) corresponding to laser and PTLS, respectively. (Scale bar, 40 μm.)

The proposed platform integrates both on-chip nanoscopy and a highly sensitive QPM system. On-chip nanoscopy offers high-throughput imaging by decoupling excitation and emission path, whereas the PTLS in QPM offers nanometric spatial phase sensitivity to identify nanometric morphological changes in the specimens. We first characterized the system by calculating the spatial phase noise (i.e., the spatial phase sensitivity of the system in QPM mode). To measure the phase noise in the system, a standard flat mirror of surface flatness λ/10 was used as an object to capture interferometric images. Fig. 4*A* shows the recorded interferogram on the mirror surface when operating the system in QPM mode. Ideally, the calculated phase map without any sample on the flat surface should be zero. However, spatial noise is always present in any QPM system, which can be difficult to avoid due to experimental imperfections such as unwanted vibrations or temperature fluctuations. Fig. 4*B* depicts the SD of the phase variations (i.e., the spatial noise of the system). The average spatial noise of the system is ±20 mrad, which is significantly less than using a direct laser to perform QPM (18, 47). Fig. 4*C* depicts the temporal noise of the phase microscopy system. To measure the temporal phase stability as shown in Fig. 4*C*, a time-lapsed movie of interference was acquired for 60 s by placing standard flat mirror. The average temporal phase stability is ± 38 mrad.

High spatial and temporal coherence of a direct laser causes speckles and spurious fringes in the final image, reducing the phase sensitivity of the QPM system. This unwanted noise can be avoided by introducing spatial and temporal diversity in the laser beam by passing it through a rotating diffuser and, subsequently, a MMFB (18). The rotating diffuser and MMFB reduce the spatial coherence of the light source, thus improving the spatial phase sensitivity of the system.

A Fourier ring correlation (FRC) test is performed on the *d*STORM data to estimate the resolution of the system in nanoscopy mode, with the result plotted in Fig. 4*D*. The resolution is given by a normalized cross-correlation between the *d*STORM images of same region in the frequency domain. To this end, the frequency spectra of two images are divided into bins to produce a series of concentric rings. The correlation value for each bin was used to form the FRC histogram. Mathematically,

[4] $$\mathrm{FRC}\left(r_{i}\right)=\frac{\sum_{r\in r_{i}}FT_{1}\left(r\right)\;.\;FT_{1}(r)}{\sqrt{\sum_{r\in r_{i}}FT_{1}{}^{2}\left(r\right)\;.\;\sum_{r\in r_{i}}FT_{2}{}^{2}(r)}},$$

where $FT_{1}\left(r\right)$ and $FT_{2}\left(r\right)$ represent the Fourier transform of the two images of same region. The image resolution is defined from the cut-off frequency at which the cross-correlation drops below the preset threshold value. In our case, we first separated odd and even frames of the acquired datasets, reconstructing them separately to generate two *d*STORM images of same region, which provide a value of 61 nm according to the FRC analysis. The resolution of our system can, however, be increased by swapping the beam splitter (marked BS in Fig. 2) in the system for a flip mirror, as half of the photons are lost passing through it. We chose to use a beam splitter, as it opens up for simultaneous fluorescence and phase imaging, whereas a flip mirror would limit the setup to sequential imaging.

In addition, to explain the advantage of partially spatially coherent source, we show the comparison between conventional coherent QPM and partially spatially coherent QPM systems to recover the phase map of an optical waveguide. The experiment is conducted on a rib optical waveguide with a core material of silicon nitride (Si_3_N_4_), refractive index n ∼2.04, and with a rib height of ∼8 nm. Fig. 4 *E* and *F* depict the interferometric images of the waveguide sample of 8-nm step height using coherent and partially coherent light sources. Here, the difference in the fringe quality generated by the laser and the PTLS is evident. The reconstructed phase map of the step object (waveguide) is shown in Fig. 4 *E,* 1 and *F,* 1. The object structure is not reconstructed by the coherent source due to the presence of coherent noise. Contrary to this, the phase recovery of 8-nm step height object can be seen clearly obtained by the incoherent illumination (Fig. 4 *F,* 1). This result further highlights the advantage of using a PTLS to recover the phase map of thin specimen.

**Fig. 5.**
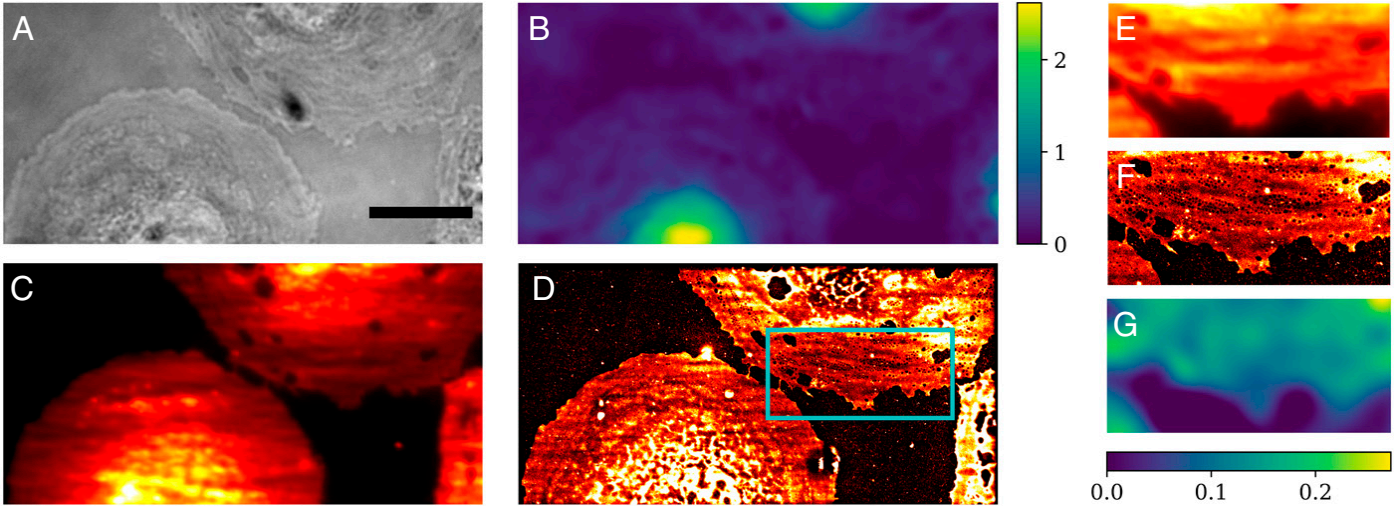
Parts of three cells imaged with bright field (*A*), QPM (*B*), TIRF (*C*), and *d*STORM (*D*). (Scale bar, 10 μm.) The phase map gives morphological information about the cells, with a maximum phase value in the nucleus of the lower left cell of 2.3 rad. The *d*STORM image clearly shows plasma membrane fenestrations in the upper cell. TIRF, *d*STORM, and QPM images of the inset in *D* are also respectively presented in *E*–*G*. The color bars show phase in radians.

Fig. 5 shows a complete dataset gathered for one imaged region of LSEC. It consists of 1) the bright-field image, 2) the phase map of the LSEC, 3) the diffraction-limited TIRF image, and 4) the *d*STORM image with visible fenestrations. The bright-field image (Fig. 5*A*) offers clear diffraction-limited qualitative imaging of the cell. On the other hand, Fig. 5*B* represents a quantitative phase map (i.e., optical thickness of the LSEC). In the phase image, the higher phase region in deep yellow represents the nucleus surrounded by the plasma membrane. The maximum phase value is 2.3 rad in the nucleus of the bottom left cell. Fig. 5*C* represents a diffraction-limited TIRF image of the LSEC, which offers excellent optical sectioning, showing morphological and functional features of the cells. However, visualization of fine features, such as fenestrations present in the plasma membrane (Fig. 5*D*), can only be attained when using superresolution imaging. Fig. 5 *E*–*G* present the inset from Fig. 5*D* in TIRF, *d*STORM, and QPM mode. Comparing Fig. 5 *E* and *F*, the fenestrations in the membrane resolved in the *d*STORM image are not visible using diffraction-limited TIRF imaging.

In addition, to include the validation and more statistical analysis, several experiments were performed on a total of five different batches of mouse LSEC. To demonstrate the possibility of precise detection of changes in the cell height, samples were treated with cytochalasin B (10 μg/mL). This actin cytoskeleton disrupting agent has been studied extensively on LSEC with well-defined effects. Cytochalasin B was shown to increase the number of fenestrations in LSEC as well as decrease the cell height distal to the nuclear area (36).

**Fig. 6.**
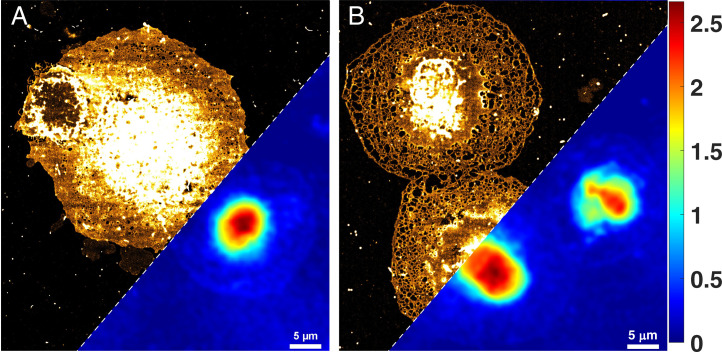
(*A* and *B*) Multimodal imaging of the control and treated (cytochalasin B) LSEC using the integrated on-chip nanoscopy and high spatially sensitive QPM system. Cytochalasin B increased the number of fenestrations in LSEC as well as decreased the height outside of the nuclear area, rendering the cells flatter and more fenestrated compared to the control cell. (Scale bar, 5 μm.) The phase shows a maximum phase of 2.5 rad in the nucleus of the cell. The color scale shows phase in radians.

Both control and treated LSEC were imaged using the integrated microscopy platform and are shown in Fig. 6. Fig. 6 represents the *d*STORM and phase image of control and treated LSEC. The difference in porosity between control and treated cells can be clearly seen in the *d*STORM image. The cell height in the nuclear area remains similar for both groups, while the height in the periphery of the cell (where sieve plates are located) is decreased. The average phase value of the sieve plates in treated cells was also found to be lower compared to the average phase value of sieve plates in control LSEC. The mean phase value of the fenestrated area in control and treated cells was 161 ± 50 mrad and 109 ± 49 mrad, respectively. Therefore, the QPM, even while being diffraction limited along the lateral dimensions, provides sufficiently accurate and useful results along the axial direction. The *d*STORM image of Fig. 6 *A* and *B* show the membrane region of the control and treated LSEC.

**Fig. 7.**
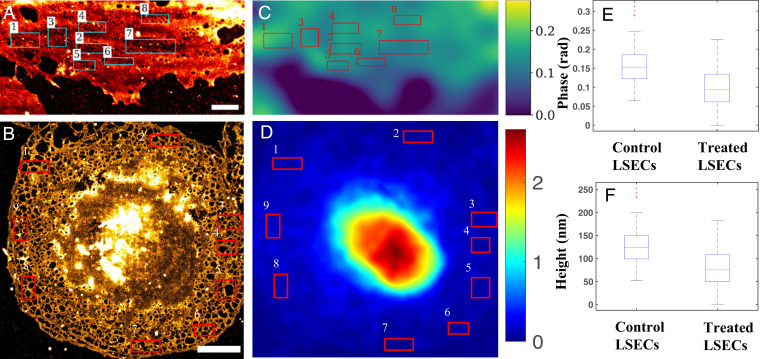
(*A* and *C*) *d*STORM and phase image of the control LSEC. (Scale bar, 2 μm.) (*B* and *D*) *d*STORM and phase image of treated LSEC. Fenestrations in the plasma membrane are visible all throughout the cell. (Scale bar, 5 μm.) The phase shows a maximum phase of 2.3 rad in the nucleus of the cell. The color bars show phase in radians. (*E*) Box plot shows average phase value of sieve plates for the control and treated LSEC. The average phase value of the fenestrated regions for control and treated cells were 161 ± 50 mrad and 109 ± 49 mrad, respectively. (*F*) The average thickness of the fenestrated region for normal treated cells was 136.6 ± 42.4 nm and 92.36 ± 41.6 nm, respectively, by assuming constant refractive index (*n* = 1.38) of the LSEC membrane. The lower phase value for the treated cell can be explained from the *d*STORM image (i.e., more fenestrations in the LSEC and, therefore, less scattering from the sample, which reduces the average phase of the treated LSE).

Fig. 7 shows the measured phase value for several different fenestrated regions of normal and treated LSEC. Although the fenestrations are below the diffraction limit and thus the spatial resolution limit of the QPM (Fig. 7 *C* and *D*), the average phase of the sieve plates can be calculated. The average optical thickness of the sieve plates can be calculated based on the phase map. A box plot of the mean phase value of sieve plates is shown in Fig. 7*E*. A total of 85 and 72 groups of fenestrated regions from 23 control and 21 treated cells, respectively, were used to show the average phase value. Considering the constant refractive index (*n* = 1.38) throughout the membrane of the cell (48, 49), the average thickness of fenestrated areas in normal and treated cells was 136.6 ± 42.4 nm and 92.36 ± 41.6 nm, respectively. The average thickness computed using QPM phase maps is an approximate value, since we assumed the constant refractive index (*n* = 1.38) throughout the membranous part of the cell (48, 49). The multimodal microscope provided an estimate of the fenestration diameter of 119 ± 53 nm (using the Sauvola local thresholding algorithm in FIJI) and an average thickness of the sieve plates of 136.6 ± 42.4 for the control LSEC.

The change in thickness can be explained, since the effect of cytochalasin B is an increased number of fenestrations in LSEC as well as a decrease in cell height distal to the nucleus, making the cells flatter and more fenestrated compared to the normal cell. The cytochalasin effect results in a more fenestrated membrane, which is clearly visible in the *d*STORM image. On the other hand, the average phase value of the fenestrated region was found to be lower for the treated cell compared to the control LSEC. The lower phase value for the treated cell can be explained from the *d*STORM image (i.e., more fenestrations in the LSEC and, therefore, less scattering from the sample, which reduces the average phase of the treated LSEC). These results are in agreement with previous studies using atomic force microscopy (36), in which a decrease in cell periphery height of about 50% was also observed.

## Conclusion

In this work, we developed an integrated multimodal chip-based optical nanoscopy and highly spatially sensitive QPM system. To demonstrate the potential of the proposed system, we localized plasma membrane fenestrations in LSEC using *d*STORM and then measured the thickness of the fenestrated areas using QPM. The system, when operated in the *d*STORM mode, offers nanometric spatial resolution (61 nm) to visualize small fenestrations present in LSEC. In the proposed system, the same imaging arm is used to capture the phase and superresolution image without any mechanical displacement in the sample; therefore, the same location of the cell can be easily identified. On the other hand, finding the same cell in two different microscopes might be challenging and is time consuming/impractical, as the size of the waveguide chip can be very large (i.e., 25×25  mm). In addition, angular displacement in the final acquired datasets using two different microscopes will certainly create a subpixel mismatch hard to avoid by image registration mechanics and, therefore, affecting the measurement accuracy of the study. Common optomechanical components were used in the imaging arm for both QPM and *d*STORM mode, whereas using two different systems will cost almost double, which can be considered as another advantage of the system described here.

The proposed system enables multimodal imaging in a simple manner while still being easy to further customize. For improved resolution in *d*STORM, a simple flip mirror, instead of a beam splitter, will help toward a better signal and, therefore, localization. Further enhancements in phase sensitivity are possible by replacing the partial spatial coherent illumination with a perfectly incoherent light source such as white light or an LED. The white light source offers maximum possible spatial phase sensitivity but requires multiple frames (i.e., phase-shifting interferometry [PSI]) to extract the phase information due to poor temporal coherence. Additionally, PSI can also be useful to improve the transverse resolution of the system. Moreover, with minor modifications, different modalities can easily be added to the current system, such as waveguide-based optical trapping (50) and spectroscopic techniques (51). There is also potential toward significant reduction of the system footprint, along the lines of automated coupling for the sake of ease of use (52). Chip-based microscopy has also been demonstrated for live-cell imaging of delicate cells (53). In the future, we aim to adapt the proposed multimodality microscopy platform for imaging dynamics of fenestrations in living LSEC (i.e., when challenged by chemicals or drugs that alter fenestrations and sieve plates). Being able to obtain both the fenestration diameter and sieve plate thickness will make it possible to track changes in a very detailed manner. This will be a particularly useful tool for the discovery of agents that reverse age-related pseudocapillarization, since the method simultaneously measures two important parameters, LSEC thickness and fenestration, that are increased and reduced, respectively, during the aging process.

## Data Availability

Processed *d*STORM images and raw interferograms that support the results within this paper are available at DataverseNO: https://dataverse.no/citation?persistentId=doi:10.18710/AWRGH1.
